# Suits for Malpractice

**Published:** 1872-07

**Authors:** H. F. Montgomery


BUFFALO

### Medical and Surgical Journal.

VOL XI. JULY, 1872. No. 12

### Original Communications.

ART. I.—*Suits for Malpractice.* (President's address to Monroe County Medical Society.) By H. F. Montgomery, M. D.

In accordance with a requirement of the By-Laws of this Society, the presiding office before retiring, delivers a discourse upon some subject pertaining to the art which we practice.

The topic now selected for consideration is one of great interest to every member of our profession. I allude to the prosecution of medical men for so called malpractice, and to the testimony of physicians *against* the defendants.

In looking over the older law reports such a case is rarely found; of late they have become frequent. At nearly every sitting of the court one or two such cases are on the calender. It is, too, a remarkable fact that the trials generally result adversely to the surgeon. It has become so great an evil that a remedy should be found. The evil and injustice to the surgeon is great, after weeks of anxiety and labor over an intractable case, the deformity is not removed, and with all his skill and care, cannot be removed. Then follows a long and harrassing trial for damages at a great cost and loss of time, however the suit may be determined.

There is the further evil that the indigent are deprived of the gratuitous services of the surgeon of experience and skill, the reason is obvious; to the ignorant, the failure to restore the injured limb to its original perfect condition is evidence of want of care, or want of skill. Acting on this idea, the unfortunate subject on an imperfect recovery, commences suit. What surgeon knowing the result of certain recent trials would have the temerity to undertake the treatment of a pauper for a compound oblique fracture of the tibia and fibula near the ankle joint? The reason for the increasing frequency of these trials is the almost uniform pecuniary success of the undertaking. Then comes the inquiry, why this successful issue to these trials? and what is the remedy? It is not the fault of the law. The principles of law upon which these suits are brought are sound and just. The great difficulty and main cause for the unjust termination of these cases is that the judges do not clearly understand the nature and character of these injuries, or the difficulties of the diagnosis and treatment. This ignorance on the part of the court arises from the carelessness, or bias, or other fault (perhaps want of candor) of medical witnesses. The law does not hold a professional man responsible for an error of judgment, but for gross ignorance or for carelessness. A member of the medical profession who has a diploma from a recognized Medical College, or whose reputation and standing in his profession is good, is not grossly ignorant. If such a person gives his best efforts and care to any given case he is not responsible for the result, whether it is good or bad. He does not agree when he takes charge of a case to be equal to the best or highest in his profession, but with ordinary skill and care to give his best efforts to relieve his patient, and having done this he is not responsible. The highest skill is not attained or attainable by knowledge or learning, or by experience even, but it is the gift of the few, it is genius. This is not only true in surgery, but in all the arts. The largest portion of laborers in any calling or profession are not distinguished for anything except for their want of distinction.

He who is the subject of injury, and who living remote from the great centres, wishes surgical aid, if he wants the best, must send *away* for it. Such a man, who with a broken or dislocated limb sends for a professional man in his neighborhood, knows that by sending further and paying more he can have the attendance of surgeons of greater skill, of men celebrated for their skill. This only the few can afford. He sends for his family physician who gives him honestly and fairly his best skill and care. The case may recover with deformity, even great deformity, and admitting that it might have done better in more skillful hands, the medical attendant is not and ought not to be liable to damages for the result. The fact that the result was not favorable, is not even presumptive evidence of want of skill or want of care, but on the contrary it is presumptive evidence that there was something obscure and only discoverable by the highest skill or by a post-mortem examination or that the difficulty was irremediable. The plaintiff must prove his case, that is, he must prove ignorance or carelessness on the part of the defendant, and if he does not, the judge ought to grant a non suit. If the plaintiff does not show that the defendant was guilty of gross ignorance or carelessness, he has not made out his case and there is no question of fact for the jury. In fact, in these cases no question ought to be submitted to the jury, until gross ignorance or carelessness has been established to the satisfaction of the court No professional man can practice with safety to his purse or reputation under any other rule of law. The propriety of his practice in detail, whether he did this or that or whether he did not, is not a question for a jury, who are entirely ignorant of the whole subject, to determine. If the court could distinctly understand, that when a surgeon comes to examine an injured limb, it was not a matter of knowledge that was to determine the extent and nature of an injury, but an opinion or judgment only, then the court would know whether or not the plaintiff had established his case.

Neither the lawyers, nor the judges, nor the jury, nor the patients understand this. They think that the surgeon's knowledge of anatomy enables him to know and demonstrate the exact amount and character of the injury. In this they are in error. The surgeon examines an injured limb or joint covered with muscles, skin and fat; into these substances there has been, by the time the surgeon sees it, blood and serum poured out to a greater or less extent, and the parts have become extremely sensitive. The surgeon cannot see the injured bone or joint, and covered as it is, with a cushion such as I have described, he can not distinctly feel the part. He can observe deformity and perhaps detect crepitation, and perhaps not. It is not a matter of knowledge, but of opinion, what is the character, nature and extent of the injury, and what is best to be done under all the circumstances. There may be fracture of the bone which produces the deformity, or the deformity may be the result of a dislocation, or the deformity may be the same in appearance or to the touch without either fracture or dislocation; as it may be produced by the effusion of blood and serum and rupture of soft parts. Which of these it is, is not a matter of demonstration but of opinion formed from many circumstances, often very occult and impossible to determine with certainty. To err in the conclusion to which the surgeon may come is not gross ignorance nor carelessness, though some other surgeon might have judged more correctly. There may be a fracture and a surgeon of skill may not find it. There may be dislocation and a surgeon of skill and experience may not detect it. This being admitted (as it will be by every surgeon of experience) a bad result is not evidence of ignorance or carelessness. In any case, when there is a difference of opinion between professional men of good and respectable standing as to the nature of the injury or as to the particular treatment, then the surgeon, charged with want of care or skill, is not responsible and the plaintiff has not established his case and should be non-suited. Another reason for the unfortunate result of these trials is the want of care and caution on the part of the medical witnesses. In giving their evidence as experts, they often give opinions without sufficient facts upon which to form an opinion and speak more confidently than the facts will warrant. They also in testifying to treatment define their own peculiar treatment as the only correct one, and any deviation from that as bad. This conveys the impression to the judge and jury of a certain fact instead of an uncertain opinion. I will endeavor to show the correction of the above conclusions by a partial report of a recent trial.

Dr——has three times been defendant in a suit for damages for not permanently restoring a dislocation at elbow joint. He attended the patient five days and then discharged himself, leaving the patient in the hands of the family physician, a homeopathist. When Dr——first saw the case he pronounced it a dislocation of both bones of fore-arm backward and as he supposed reduced the displaced bones. After several weeks the bones were found not to be in place and the patient called in another surgeon and the bones are not now in place. On the first and second trial the plaintiff undertook to show that by want of skill or care Dr—had failed to reduce the dislocation and that it had not as defendant claimed been re-dislocated after he left it.

By all the medical witnesses the plaintiff showed that re-dislocation of both bones of the fore-arm backwards, where not complicated with fracture, was very rare, so rare that most of the medical witnesses had not seen or heard or read of such a case, and yet on the third trial of this case the plaintiff's attorney changed his ground and proved by the same witnesses, that re-dislocation was so probable and so much to be feared that the omission to put a sling upon the patient's arm when lying in bed with the arm and fore-arm upon a pillow, was want of care, and the reason why it had become displaced again.

Is it not obvious that an arm and fore-arm imbedded in a feather pillow with the fore-arm at a right angle, with the arm when the patient is in bed and the pillow at his side, is more perfectly at rest and less likely to move than it would be restrained merely by a sling? Let him who doubts try it.

On the second trial one of plaintiff's medical witnesses is reported to have testified, folio 701–708. "I don't believe it was dislocated a second time" (would it have any different appearance after re-dislocation than after the priminary dislocation?) same witness, "it was put in this position where it could not have got out, without a fracture." At folio 193 the same witness testifies as to re-dislocation. "It is the most impracticable thing of any of the joints that are endowed with as much motion as that," again at folio 212 the same witness testifies in answer to the question, "if the patient was going to bed would you use a sling?"—"yes sir." At folio 235 same witness. "The elbow joint, is a joint that is so little likely to get out that, the surgeon don't think it necessary to use a splint." Another witness testified for plaintiff, folio 259; "I will premise by saying that it (re-dislocation) is not a very common thing in this joint, but it does occur. Folio 417, 418, same witness in answer to question, "would you regard a sling to be necessary?" "not positive.

ly so." 423, Question: "Is it liable to get out of that position, that angle?" same witness answered, "certainly, there is no objection to it,"(the position upon the pillow,) "if the patient would keep very still and keep it there." The judge in refusing to grant a non-suit said, folio 326, "Assuming that he did set that arm, he did not as I understand the physicians here to say that he should have done, put it into a sling." One of plaintiff's witnesses, folio 254, testified that when he saw plaintiff and examined the arm, several weeks after the injury, that he was not able to tell whether this dislocation had been complicated with fracture or not. On the third trial this same witness testified, folio 411–412 in answer to a question, "could you at that time (when he examined the arm several weeks after the injury,) have discovered whether there had been a fracture of the coronoid process?" "I think I should have been likely to have discovered it, though I know very little about that."

This witness testified on the second trial, folio 269, that in case of fracture of coronoid process, "a careful examination of it would discover this grating which is called crepitus," and that that discovery would be apt to be made by a skillful physician.

One of the witnesses for the defence, folio 319, testified that when the bones are reduced, the joint retains its position generally, if it is kept quiet. Another witness for defence, folio 569, to the question, "what do you say in relation to putting it in a sling" answered, "If the intensity of the inflammation was excessive, and the person was going to lie on their back in bed, the best position would be to put it as Dr—— did, on a pillow, *** as long as the person is lying in bed, I think the position on the pillow is the easiest for the patient, and there is no voluntary motion in a part that is painful, the pain in moving it would induce the patient not to move it." Folio 583, same witness, "I think after a simple dislocation, subsequent dislocation of it is rare." Folio 577, same witness as to fracture of coronoid process, question: "Can the parts be brought into contact so as to discover crepitus?" "I infer not always."

I give the above extracts from the printed report of the trial as specimens of the evidence. On the first trial the jury did not agree, on the second and third the verdict was in favor of plaintiff. There was so decided a difference of opinion upon the main point in this case among the medical experts, that I wrote the following letter to six distinguished professors of surgery. Five of them did me the favor and courtesy to answer my letter, and have thus placed me under obligations to them. I will state the question and the several answers:

(Copy of Letter.)

ROCHESTER, N. Y. April, 1870.

DEAR SIR: At a recent trial for malpractice in this state, the case turned upon the answers to the enclosed questions. Can you give me your answer to these questions? I wish by you to confirm or not my evidence given upon the trial.

Signed, Yours truly,

H. F. MONTGOMERY, M. D.,
Surgeon Rochester City Hospital.

QUESTIONS.

1st. In a case of dislocation backward of both bones at the elbow joint, not complicated with fracture, the result of a fall from a runaway horse, in a female, would it be proper treatment, immediately after having reduced the dislocation, to place the patient on the back in bed, with the arm and fore-arm upon a pillow at her side, with the fore-arm at about a right angle with the arm?

2d. Would it be necessary upon such a patient so placed to put a sling around the neck and fore-arm, to prevent a re-dislocation?

The above letter and questions were sent (with the names of the following professors of surgery) to each of these gentlemen:

To Prof. FRANK H. HAMILTON, M. D., New York.
" " HENRY H. SMITH, M. D., Philadelphia.
" " WILLARD PARKER, M. D., New York.
" " ALFRED C. POST, M. D., New York.
" " THOMAS M. MARKOE, M. D., New York.
" " JAMES H. ARMSBY, M. D., Albany.

Below I give the five answers received from five of the six gentlemen to whom I wrote. These answers are *not* given in the order of the above list, as I have not had their consent to this publication.

Copy.

April, 1870.

DR. H. F. MONTGOMERY, Rochester City Hospital.

DEAR SIR: In reply to your note of 14th inst. to day received, asking my opinion on the after treatment of luxation of both bones of fore-arm backwards. I would state that I am unable to reply to a special case, as swelling, &c., *might* render confinement in bed with the arm flexed on a pillow appropriate treatment. But unless there was contusion tumefaction &c. I generally apply a roller *lightly* from fingers to middle of arm or higher and then an *angular* splint (flexion) to front of fore-arm and arm as in fracture of condyles, making passive motion subsequently.

Respectfully Yours.

———————

Copy.

April, 1870.

MY DEAR SIR: In a case such as you describe, of dislocation of the elbow in an intelligent adult, who understood the importance of keeping the joint quiet during the healing of the lacerated structures, I should think that the position you describe would be quite sufficient as a plan of treatment to secure a good result. This I should think would be particularly the case with the elbow in which joint, from its ginglymoid character, re-dislocation would not easily be produced. In a child or a restless intractable patient of any age I should think a sling very important.

Very respectfully your obedient servant

Dr. H. F. MONTGOMERY, M. D. ———————

(Copy.)

April, 20th, 1870.

H. F. MONTGOMERY, M. D.

DEAR DOCTOR: In answer to your first question I reply that in the first place a patient is not necessarily confined to bed after reduction of dislocation of the elbow; and secondly if the patient be placed in bed the fore-arm and hand should be placed across the chest rather than by the side of the patient. The fore-arm should be bent at a right angle with the arm. In answer to your second question I reply that the fore-arm and hand should by all means be supported by a sling even in the recumbent posture, especially during the hours of sleep. An intelligent person, when awake might be trusted without the sling while in the recumbent posture.

But if he should fall asleep without the support of the sling, he might make some unlucky movement which would endanger a reproduction of the dislocation.

Yours sincerely.

———————

Copy.

April 11th, 1870.

DEAR DOCTOR: I have received yours, and in reply I have simply to say that when the bones are reduced the fore-arm must be maintained in nearly a right angular position with the arm.—2d. This indication is met by resting the arm and fore-arm upon a pillow when in bed, and by supporting the fore arm and hand in a sling when sitting up or standing, and hence the sling is unnecessary while lying in bed.

Yours Truly

H. F. MONTGOMERY. ———————

Copy.

April 17th, 1870.

DR. MONTGOMERY.

DEAR SIR: As you will see by reference to interrogation, No's. 1 & 2, I have answered them respectively yes to No. 1. and no to No. 2. I may add that having reduced such a dislocation, I ordinarily adopt no precaution to prevent a re-dislocation. I will not deny the possibility of its becoming spontaneously displaced, but if it does occur it is so rare that it has never happened to me, although I have reduced a great many in my life.

Very Truly Yours,

———————

It will be observed that these distinguished teachers of modern surgery are nearly equally divided upon this question of the use of a sling in bed. Three of them answered the question as did the medical witnesses for the defence and two were with the witnesses for the plaintiff. Is it not preposterous, to ask a jury of twelve farmers to determine which of these learned and experienced professors are correct in their practice. Its absurdity seems to be apparent. If this decision of the court is sustained by the court of errors, where the case is to be argued, it will not be safe to practice any profession, much less surgery. The plaintiff did not show by the evidence that Dr—was greatly ignorant nor did they show him careless—on the contrary the evidence showed the extreme of care, for he called in counsel to see the patient twice during the five days that he was in charge of the case. He told the patient the first time he saw the case what the injury was, and he reduced the dislocation as he supposed and believed. If he was mistaken it was no more than has happened to surgeons of greater fame and of larger experience. If it became displaced after he left it, he cannot be called upon to show how or why that occurred. It might have been from fracture of the coronoid process or from some complication unknown and not discoverable during the life of the patient. Lord Mansfield the ablest chief justice of the King's bench used the following language as to the responsibility of professional men. "Attorneys who conduct themselves with honor and integrity ought to be protected, when they act to the best of their skill and knowledge. Every man is liable to errors and I should be very sorry to think that it should be taken for granted that an attorney is answerable for every error or mistake and to be punished for it by being charged with the debt he was employed to recover."

In the case of Percy v. Milloudon, Louisiana Reports No. 8, New Series.—Porter J. remarked: "It has been said that it will not be sufficient for a professional man to say he acted to the best of his abilities, because he should have formed a more just estimate of his own capacity before he engages himself. This doctrine, if sound, would make an attorney responsible for every error of judgment, no matter what care or attention he exercised in forming his opinion. It would make him liable in all doubtful cases, where the wisdom or legality of one or more alternatives was presented for his consideration, no matter how difficult the subject. But when a person who is appointed an attorney has the qualifications necessary for the discharge of the ordinary duties of the trust imposed, we are of the opinion, that the occurrence of difficulties in the exercise of it, which offer only a choice of measures; the adoption of a course from which loss ensues, cannot make the agent responsible if the error was one into which a prudent man might have fallen. The contrary doctrine seems to suppose the possession and requires the exercise of perfect wisdom. No man would undertake to render a service to another on such severe conditions."

E. Darwin Smith, Judge of the Supreme Court of this State, on an application for a new trial by Dr——gave the following written opinion.

Copy.

SUPREME COURT.—Monroe General Term.

December, 1869.

Present, E. Darwin Smith, Dwight and Johnson, Justices.

E. DARWIN SMITH, J.

"The motion for a non-suit made at the close of the evidence in this case I think should have been granted. I was strongly inclined to grant this motion at the trial, but thought the jury might probably find for the defendant, which would end the litigation. The principles governing the case were I think in substance correctly stated to the jury."

"There was no basis for a recovery on the ground of malpractice. On the question, of negligence upon which the case was submitted to the jury, there is not I think, upon a careful consideration of the question really any sufficient evidence to warrant the verdict. The defendant, as the proof shows, clearly possessed the ordinary skill of his profession and exerted such skill to the best of his ability. He attended the plaintiff in an emergency, and attempted to reduce the dislocation after several other physicians of the village where he resided had refused to do so. He did his best to reduce the dislocation and supposed he had effectually done so. He gave the proper directions in regard to the patient and left. He visited her the next day with Dr—— another physician and surgeon of the same village, when they both made examination and found and decided that the dislocation had been reduced and the bones of the arm were in their proper places. He visited her again after a day or so and saw her often afterwards. If the dislocation was not in fact reduced in the first instance, it is quite clear that the defendant supposed and believed it was so reduced and so also did Dr—— who examined the arm carefully the next day, after the dislocation and the attempted reduction and said at the time that the bone was in place. If there was mistake on this subject, it was not the result of a hasty inconsiderate attempt or opinion of the defendant. He clearly exercised his best skill, care and judgment, what more could he do? what more could be required of him? The call to his aid and advice of Dr.——an older physician of forty years practice, the next morning confirms this view and shows that he was anxious to do his duty and do the utmost that his skill and knowledge and efforts could effect for the plaintiff. Under such circumstances I cannot think the defendant was responsible for the consequences resulting to the plaintiff from this dislocation. He was not and is not an insurer, such was not his contract. He did not guarantee a cure or undertake that the plaintiff's arm should be restored to its former state of soundness. No man can safely practice surgery, or medicine, if a surgeon can be made or held liable to respond in damages in a civil action for a failure to restore a limb or to effect a perfect cure under such circumstances. The contract of a physician and surgeon is that he possesses the ordinary skill of his profession and that he will exercise and use such skill to the best of his judgment and ability, and with due care to ensure the cure of his patient. When he has done this his duty is fulfilled. A physician and surgeon should not be liable and mulcted in damages except in a clear case of malpractice and gross negligence, and negligence should not in such cases be implied or imputed upon the slight or uncertain evidence which is often applied in suits against corporations or wrong doers. Physicians and surgeons practice their profession under circumstances of great trust, painfulness and responsibility, and it is not right to super-add to this burden of care and duty and danger, the peril of a law suit with the right of recovery for every mistake or error of judgment which they may, and must unavoidably commit. The law is not so unjust and so unfriendly to the medical profession. I think there should be a new trial with costs to abide the result."

E. DARWIN SMITH.

The above opinion is all that we could ask of the court, and yet upon the third trial the presiding judge refused to grant a non-suit and for the second time the jury rendered a verdict against the defendant for large damages. It is to be hoped that a higher tribunal (before which this case will soon be brought) will give to surgeons the benefit of the same just rules laid down by Lord Mansfield and Judge Porter as applicable to attorneys.

We have however a remedy which depends upon ourselves. We can arrest this growing evil, if we weigh our evidence and not be led to emphasize it on this side or that as the attorneys in the case may wish. We ought to instruct the court that surgery is not an exact science nor a perfected art. That deformity more or less is the rule after fractures and frequently after dislocations, and that as to the detail of practice in any given case the surgeon in attendance must be the judge as to what is best to be done. He only can know the condition of the patient and form any correct opinion of the extent and nature of the injury to the tissues, and how much and how little pressure they will bear. That inflammation and swelling may be so extreme that a bandage of any kind would be detrimental and might even produce mortification of a part or of the entire limb is known and admitted. The medical expert should have a constant and sensitive regard for the reputation of his professional brother. If we expect the confidence of the public we must command it by our respect for each other. Especially should we guard against the natural tendency to magnify our own peculiar opinions. In giving an opinion from the witness stand we should insist upon stating when ever it could be done with truth, that though the opinion given is decided, yet that a different opinion or plan of treatment is held or pursued by able men in the profession. We should further instruct the court and jury that no two cases of disease or injury are precisely alike, and that writers on surgery or medicine in giving the treatment for any case or class of cases only undertake to give the general principles of as applied to ordinary cases, and that exceptional cases are of daily occurrence. Since this paper was first written this case has been carried to the Supreme Court on a bill of exceptions for a new trial. The application was denied. The following extract from the opinion of the court (Mullen P. J.) shows how difficult it is to make a lawyer or the court understand a surgical case, and how imperfectly the opinions of the profession are brought out before a court and jury.

Opinions of MULLEN P. J. extract from.

"The Surgeons disagreed as to the necessity of putting the arm in a sling after the dislocation is reduced, some insisting that it is necessary in order to prevent a re-luxation which might occur if the arm was left without using this means of preventing it, while others insist that it is enough to leave the cure to nature, the surgeon merely applying or directing the application of cold water to the limb in order to keep down inflammation. The defendant did not use a sling and it was for the jury after weighing the reasons assigned by the surgeons, for and against the use of it to say, whether it was negligence in the defendant to omit it."

"The defendant's counsel insist that, as it is shown, that surgeons do not agree in regard to the propriety of the use of the sling, the jury were not at liberty to find there was negligence on the part of the defendant in omitting it. I cannot assent to this proposition thus broadly stated. If writers on the treatment of dislocations, or, if in the absence of such authority, practical surgeons prescribe a mode of reducing them, and of treating them, and of treating the joint after the bones are replaced, it is incumbent on surgeons called to treat such an injury to conform to the system of treatment thus established, and if they depart from it, they do it at their peril."

"In 2d Espinassis N. P. 601 it is said, it seems that any deviation from the established mode of practice, shall be deemed sufficient to charge the surgeon with negligence in case of an injury arising to the patient. If, however, it is shown that surgeons have applied a different system of treatment and found it to succeed as well or better than the one prescribed, it is not negligence to resort to the system, thus practically tested. But before the new practice can be used to shield the surgeon from the charge of malpractice, it must appear that the cases in which it was tested were substantially the same as those treated of by the writer or with those treated by practical surgeons, and that the treatment thu resorted to has been successful in so many instances as to establish satisfactorily the propriety and safety of adopting it. The question is as a general rule exclusively for the jury and in this case it was peculiarly so.

If in case of dislocation of the elbow joint it is enough for the physician to replace the bones and to put the arm on a pillow with the part below the elbow joint at a right angle with that above it, and directing the application of cold water, it would seem to be proper if not necessary that the attending surgeon should inform the patient or those having charge of him or her, of the necessity of maintaining that position, and if there is a tendency in the limb to become straight, or if in consequence of the severity of the injury to the ligaments about the joint, there is great pain which renders the patient nervous and restless, thus increasing the tendency to re-luxation or to straighten, and as a consequence to stiffen the joint, the danger should be disclosed, to the end that all proper precaution may be taken to prevent it. The defense insists that these dangers were emminent and yet no word of warning was given. This was in my judgment culpable negligence; much of the suffering the plaintiff has undergone, and much of the loss he has sustained, might have been prevented, had the defendant done what it was clearly his duty to do, if he knew the consequences which might result from re-dislocating the joint or straightening the arm. It would seem to me that a sling would have in some degree mitigated if not altogether prevented the misfortune which has befallen the plaintiff."

The honorable and learned judge has much to say about authorities, and yet ignores the fact that the medical witnesses on the trial were authority, and the only authority recognized by the courts, and that in fact the practical surgeon of learning and of successful experience is better authority than or at least as good as any written authority. Placing the arm on a pillow when the patient is in bed was correct treatment according to authority and was not new treatment, and it was not shown to be the cause of the injury to the plaintiff, and consequently the case above quoted by the learned judge does not apply to this case. Several witnesses stated that the treatment was proper, and Ferguson, author of a work on Surgery, states page 117, "In dislocation as soon as reduction is accomplished the need for the surgeon has almost ceased—the act of reduction seems the chief part of his duty, and moreover it may be said that, in general it is the only occasion on which any active interference on his part is required."

The learned judge has decided that when the practice of different surgeons on any particular case differs, it is for the jury to say whether the following of one or the other plan is want of care or skill and consequently malpractice. The learned judge must be in error. It has not been so held in other professions and should not be in surgery. The learned judge argues the case as if placing an injured arm upon a pillow was unusual practice. Every surgeon knows that this position is the usual and best position for some of the severest injuries to the elbow joint or fore-arm. It is the position which preserves the member most perfectly from the least motion. Voluntary or involuntary motion of a limb so placed, whether the patient is awake or asleep, is prevented by the *pain* which motion produces and by the trough formed in the pillow by the weight of the limb. This position allows less motion of the joint than when the arm is supported by a sling in a person sitting up or walking about. The sling is directed to be used by a person standing or walking. The arm upon a pillow is the position for one so injured as to lie down or who needs perfect rest of the joint and when the swelling or inflammation renders bandages and splints injurious and painful and dangerous to the vitality of the injured limb. In such a case it is the common practice of the profession and it is the only proper practicable position. Whether any particular case is one of this character, can only be determined by the attending surgeon. If he errs it is an error of judgment and he cannot be held responsible. This case is soon to be argued before the Court of Appeals of this State.

